# Electrophysiological biomarkers of brain function in CDKL5 deficiency disorder

**DOI:** 10.1093/braincomms/fcac197

**Published:** 2022-08-04

**Authors:** Joni N Saby, Patrick J Mulcahey, Alexis E Zavez, Sarika U Peters, Shannon M Standridge, Lindsay C Swanson, David N Lieberman, Heather E Olson, Alexandra P Key, Alan K Percy, Jeffrey L Neul, Charles A Nelson, Timothy P L Roberts, Timothy A Benke, Eric D Marsh

**Affiliations:** Division of Radiology Research, Children’s Hospital of Philadelphia, Philadelphia, PA 19104, USA; Division of Child Neurology, Children’s Hospital of Philadelphia, Philadelphia, PA 19104, USA; Orphan Disease Center, Perelman School of Medicine, University of Pennsylvania, Philadelphia, PA 19104, USA; Department of Pediatrics, Vanderbilt Kennedy Center, Vanderbilt University Medical Center, Nashville, TN 37232, USA; Cincinnati Children’s Hospital Medical Center, Division of Neurology and University of Cincinnati College of Medicine, Cincinnati, OH 45229, USA; Department of Neurology, Boston Children’s Hospital, Boston, MA 02115, USA; Department of Neurology, Boston Children’s Hospital, Boston, MA 02115, USA; Department of Neurology, Boston Children’s Hospital, Boston, MA 02115, USA; Department of Hearing and Speech Sciences, Vanderbilt Kennedy Center, Vanderbilt University Medical Center, Nashville, TN 37232, USA; Department of Pediatrics, University of Alabama at Birmingham, Birmingham, AL 35233, USA; Department of Pediatrics, Vanderbilt Kennedy Center, Vanderbilt University Medical Center, Nashville, TN 37232, USA; Laboratories of Cognitive Neuroscience, Boston Children’s Hospital, Boston, MA 02115, USA; Department of Pediatrics, Harvard Medical School, Cambridge, MA 02115, USA; Graduate School of Education, Harvard University, Cambridge, MA 02115, USA; Division of Radiology Research, Children’s Hospital of Philadelphia, Philadelphia, PA 19104, USA; Department of Pediatrics, University of Colorado School of Medicine and Children’s Hospital Colorado, Aurora, CO 80045, USA; Department of Neurology, University of Colorado School of Medicine and Children’s Hospital Colorado, Aurora, CO 80045, USA; Department of Pharmacology, University of Colorado School of Medicine and Children’s Hospital Colorado, Aurora, CO 80045, USA; Department of Otolaryngology, University of Colorado School of Medicine and Children’s Hospital Colorado, Aurora, CO 80045, USA; Division of Child Neurology, Children’s Hospital of Philadelphia, Philadelphia, PA 19104, USA; Orphan Disease Center, Perelman School of Medicine, University of Pennsylvania, Philadelphia, PA 19104, USA; Departments of Pediatrics and Neurology, Perelman School of Medicine, University of Pennsylvania, Philadelphia, PA 19104, USA

**Keywords:** CDKL5 deficiency disorder, evoked potentials, quantitative EEG, biomarkers, developmental encephalopathies

## Abstract

CDKL5 deficiency disorder is a debilitating developmental and epileptic encephalopathy for which no targeted treatment exists. A number of promising therapeutics are under development for CDKL5 deficiency disorder but a lack of validated biomarkers of brain function and clinical severity may limit the ability to objectively assess the efficacy of new treatments as they become available. To address this need, the current study quantified electrophysiological measures in individuals with CDKL5 deficiency disorder and the association between these parameters and clinical severity. Visual and auditory evoked potentials, as well as resting EEG, were acquired across 5 clinical sites from 26 individuals with CDKL5 deficiency disorder. Evoked potential and quantitative EEG features were calculated and compared with typically developing individuals in an age- and sex-matched cohort. Baseline and Year 1 data, when available, were analysed and the repeatability of the results was tested. Two clinician-completed severity scales were used for evaluating the clinical relevance of the electrophysiological parameters. Group-level comparisons revealed reduced visual evoked potential amplitude in CDKL5 deficiency disorder individuals versus typically developing individuals. There were no group differences in the latency of the visual evoked potentials or in the latency or amplitude of the auditory evoked potentials. Within the CDKL5 deficiency disorder group, auditory evoked potential amplitude correlated with disease severity at baseline as well as Year 1. Multiple quantitative EEG features differed between CDKL5 deficiency disorder and typically developing participants, including amplitude standard deviation, 1/f slope and global delta, theta, alpha and beta power. Several quantitative EEG features correlated with clinical severity, including amplitude skewness, theta/delta ratio and alpha/delta ratio. The theta/delta ratio was the overall strongest predictor of severity and also among the most repeatable qEEG measures from baseline to Year 1.

Together, the present findings point to the utility of evoked potentials and quantitative EEG parameters as objective measures of brain function and disease severity in future clinical trials for CDKL5 deficiency disorder. The results also underscore the utility of the current methods, which could be similarly applied to the identification and validation of electrophysiological biomarkers of brain function for other developmental encephalopathies.

## Teaser

Saby *et al*. describe evoked potentials and quantitative EEG parameters in children with CDKL5 deficiency disorder, correlations between these measures and clinical severity and the potential for these EEG-based measures to serve as objective measures of brain function and disease severity in future clinical trials for this population.

## Graphical Abstract

**Graphical abstract:**
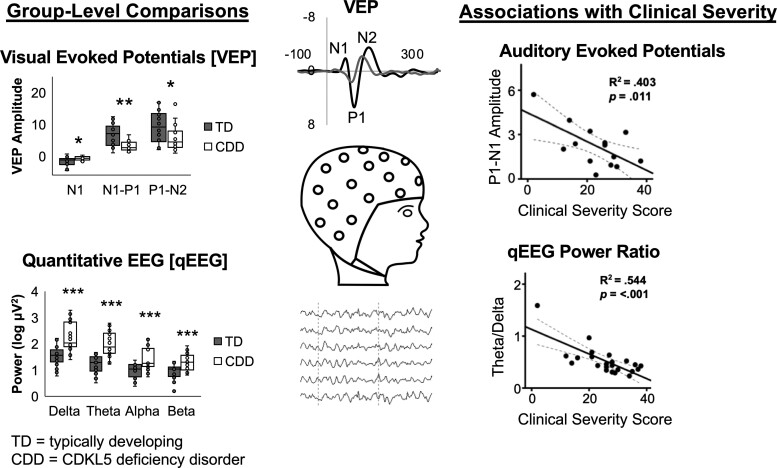

## Introduction

CDKL5 deficiency disorder (CDD) is a rare genetic condition resulting from mutations in the cyclin-dependent kinase-like 5 (*CDKL5*) gene. CDD is associated with early-onset seizures and motor, cognitive, visual and autonomic impairments that persist across the lifespan.^1,2^ CDD affects more females than males (4:1) with an estimated overall prevalence of ∼1 in 40 000.^3^

No targeted treatments exist for CDD. Anti-seizure medications are used to control seizures, with varying degrees of success, and have no impact on non-seizure outcomes.^4^ Fortunately, research advances at the preclinical level have created optimism for a better future for the treatment of CDD, including the potential for a gene therapy.^5,6^

Sensitive, robust and reproducible outcome measures to evaluate the efficacy of novel therapeutics will be needed in clinical trials for CDD, particularly since there is no precedent for normalization of function in individuals with profound neurological impairments. Recent progress has been made in the development of a comprehensive severity assessment for CDD, the first severity scale designed specifically for the condition.^7,8^ While this will advance the ability of clinicians to track the severity of CDD symptoms, there remains a need for a biological marker of severity in CDD to complement rating scales and provide an unbiased and potentially more sensitive measure of brain function in this population.

Electrophysiological biomarkers are non-invasive, easily repeated and can be acquired without the use of sedation.^9–11^ Electrophysiological measures are also translatable between animal models and humans, allowing the same outcome measures to be used at the preclinical and clinical levels of drug assessment.^12,13^ Electrophysiological parameters have been studied in conditions similar to CDD with promising results.^11,13–18^ Indeed, our recent analysis of evoked potentials (EPs) in individuals with Rett syndrome revealed an association between response amplitude and severity of Rett symptoms, underscoring the potential for these measures to serve as unbiased measures of severity in Rett syndrome.^15^ In contrast, no studies to date have examined EPs in individuals with CDD. One prior study considered aspects of the resting EEG in individuals with CDD, but statistical analyses were limited due to a small sample size (*n* = 4).^19^ The aim of the current study was to assess the potential utility of electrophysiological-based measures, specifically EPs and quantitative EEG (qEEG) parameters, as biomarkers of brain function and clinical severity in CDD. Acquisition of the EP/EEG data was intertwined with the Rett Syndrome and Rett-Related Disorders Natural History Study (NHS), a multi-site study that followed participants for up to 5 years. We present the analysis of the EPs and qEEG parameters from the CDD cohort of the NHS at the first (baseline) and second (Year 1) visits, correlate the parameters with measures of clinical severity and assess the stability of the parameters over time.

## Materials and methods

### Participants

All participants were enrolled in the NHS of Rett and Related Disorders (U54 HD061222; ClinicalTrials.gov: NCT00299312/NCT02738281) protocols 5211 and 5212. The electrophysiological data were acquired at one of five sites: Boston Children’s Hospital (BCH), University of Colorado/Children’s Hospital Colorado (UC-CHCO), Children’s Hospital of Philadelphia (CHOP), Cincinnati Children’s Hospital (CCH) or Vanderbilt University Medical Center (VUMC). The experimental protocol was approved by the appropriate Institutional Review Boards of CHOP, VUMC, BCH, CCH and UC-CHCO. For the natural history protocol (5211), the appropriate Institutional Review Boards of CHOP and VUMC approved the protocol, whereas UC-CHCO, BCH and CCH relied on the single-IRB agreement provided by the University of Alabama at Birmingham. Written informed consent was obtained for each participant according to the Declaration of Helsinki.

**Figure 1:**
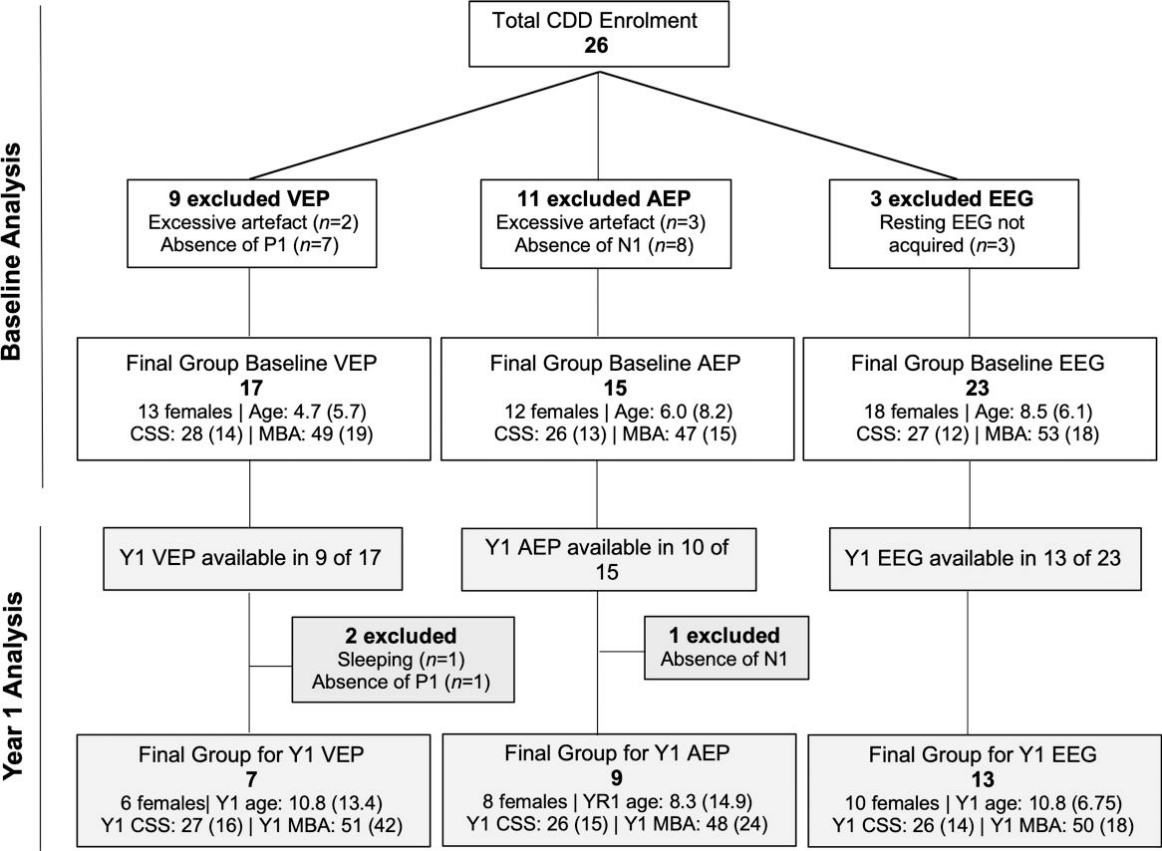
**Exclusions and demographics.** Exclusions and demographics for CDD participants included in the baseline and Year 1 VEP, AEP and qEEG analyses. Age and severity data are presented as median (inter-quartile range). CSS, Clinical Severity Score; MBA, Motor-Behavioral Assessment.

Twenty-six individuals with CDD were enrolled in the study between February 2017 and August 2021. Inclusion criteria for the CDD group included a documented pathogenic variant in the CDKL5 gene and a confirmed diagnosis of CDD made by a child neurologist.^1^ Several CDD participants were excluded from the analysis of the EPs and/or qEEG for excessive EEG artefact or other reasons (see Fig. 1 for details of exclusions). Follow-up data were available in a subset of participants who returned for a second visit. The follow-up (‘Year 1’) visit occurred, on average, 12 months after the baseline visit (range = 6–22 months). Analyses of the visual evoked potentials (VEP) are based on 17 participants for baseline and 7 for Year 1. Analyses of the auditory evoked potentials (AEP) are based on 15 participants for baseline and 9 for Year 1. Analyses of the qEEG are based on 23 participants for baseline and 13 for Year 1 (see Fig. 1 for demographics). There was no difference in the Clinical Severity Scores of participants who returned for follow-up (median = 28) and those who did not (median = 27; *P* = 0.621).

Eighteen typically developing (TD) individuals served as the comparison group (13 females, median = 5.4 years of age; range = 1.4–25.4 years). TD participants were selected from a larger pool of TD participants from the NHS to match the CDD cohort for age and gender. Specifically, all TD females under 10 years of age were selected. TD males and females >10 years of age were only selected if they matched one-to-one with a CDD participant. TD participants were pre-screened and excluded using the same criteria as Saby *et al*.^15^ Briefly, potential TD participants were excluded if they had a history of developmental delay or known neurologic, neuropsychiatric or genetic condition. Typical development was confirmed using the Child/Adult Behavior Checklist and either the Ages and Stages Questionnaire (for >5 years of age) or Wide Range Achievement Test-4 (for ≤5 years of age).^20–22^ Given an established literature on stability and change in EPs in TD individuals, we did not follow TD participants longitudinally. Data from a second (Year 1) visit were available in a small subset of TD participants (*n* = 5; 2 females; median = 3.8 years of age, range = 2.1–25.4 years). These data were used to confirm that our procedures were resulting in intersession repeatability in this group, as would be expected based on the larger literature with TD children and adults.^23–26^

### Clinical measures and variables

Two clinician-completed assessments were used to estimate overall severity: the Clinical Severity Score (CSS)^27,28^ and the Motor-Behavioral Assessment (MBA).^28,29^ These assessments were created for Rett syndrome, but account for many symptoms of CDD including epilepsy and motor, cognitive and autonomic disturbances. The CSS has 13 items with a maximum score of 58. The MBA has 34 items with a maximum score of 136. CSS and MBA scores for the participants in the present study are provided in Fig. 1. Clinical scores were typically obtained on the same day as the EPs/EEG. When visits did not occur on the same day, the scores from the closest clinical visit were used for correlating with the EP/qEEG parameters.

Several other clinical variables were considered including seizure frequency and number of medications. Seizure frequency was defined using an ordinal variable with seven categories: absent, less than monthly, monthly, weekly to monthly, weekly, daily or multiple times per day. The number of medications was the sum of all medications taken excluding keto diet and rescue medications. Additional group analyses were conducted for the presence or absence of benzodiazepines.

### EEG/EP recording

All sites followed standardized procedures for the EP and EEG acquisition. The VEP stimuli consisted of 400 trials of a reversing black and white checkerboard presented continuously (0.5 cpd, 100% contrast, 2 Hz refresh rate). One study site (BCH) employed eye tracking (Tobii Technology, Danderyd, Sweden) to automatically pause the visual paradigm when participants looked away from the stimulus. To ensure the VEP results from this site were consistent with the other sites, additional analyses were conducted on this site only (see Supplementary Fig. 1). The AEP stimuli consisted of 520 trials of 500 Hz sinusoidal tones (300 ms duration) with a varying interstimulus interval of 0.6–2 s. The tones were presented at 60 dB SPL using a free-field speaker. Resting EEG was acquired for a median of 10.45 min (range: 5.05–16.23 min) for CDD and TD participants. During the acquisition of the AEP and resting EEG, participants were permitted to view a silent movie. The order of tasks (VEP, AEP, resting EEG) was randomized between participants. A member of the research team observed the participant during the duration of the recording to ensure wakefulness and direct their attention to the stimuli when necessary.

### EEG methods

EEG equipment varied by site. At CCH, EEG was recorded from 21 individual Ag/AgCl electrodes using a Natus EEG32U Amplifier (Natus Neuro, Middleton, WI, USA; 512 Hz SR). At CHOP, EEG was acquired using a 60-channel Ag/AgCl electrode cap using the EEG amplifier of an Elekta VectorView (Elekta Oy, Helsinki, Finland; 1000 Hz SR). For the other sites, EEG was recorded from a 128-channel Electrical Geodesics Net using a Net Amps amplifier (Electrical Geodesics, Inc., Eugene, OR, USA). Electrode impedances were checked before all recordings and kept below the individual systems’ recommendations. To account for site differences in the amplitude and latency of the EPs, data for all CDD and TD participants were adjusted prior to the final analysis. Adjustments were based on data from a travelling, adult human phantom who completed the EP tasks at all study locations (for further details of equipment and correction, see Saby *et al*.^15^).

Data analysis was performed at one central location (CHOP). EPs were analysed in BESA (BESA 6.0 GMbH, Grafelfing, Germany) using methods described previously.^15^ Briefly, files collected at 1000 Hz were downsampled to 512 Hz. Bad channels and periods of excessive artefact were manually marked and excluded. Ocular artefacts were removed using automatic artefact correction methods in BESA. The artefact-corrected data were transformed to a reference-free, 81-channel array and digitally filtered using a 3 Hz high-pass filter. The continuous files were segmented into 500 ms epochs for the VEP (−100 to 400 ms relative to stimulus onset) and 600 ms for the AEP (−150 to 450 ms). The segmented files were baseline-corrected based on the mean of the pre-stimulus period and low-pass filtered at 40 Hz. Epochs were excluded if the amplitude at any channel exceeded ±250 μV. The number of accepted trials for the VEP was comparable for TD and CDD participants (TD: median = 343, range = 253–396; CDD: median = 347, range = 199–397; *U* = 137, *P* = 0.613). The number of accepted trials for the AEP was slightly lower in participants with CDD (TD: median = 482, range = 344–518; CDD: median = 430, range = 281–517; *U* = 67.0, *P* = 0.013). Analysis of the VEP focused on the N1, P1 and N2 components of the response at the midline occipital electrode (Oz). Analysis of the AEP focused on the P1, N1 and P2 components of the response at the frontal–central midline electrode (FCz). Components were identified using the automatic peak finder in BESA using predetermined criteria (see Saby *et al*.^15^).

The resting EEG files were preprocessed in BESA 6.0 and analysed in MATLAB (v2019b, Mathworks, Natick, MA, USA). Files collected with a 60- or 128-channel EEG configuration were first visually inspected for bad channels and periods of excessive artefact, which were excluded from further analysis, and then reduced to a 10–10 virtual montage to allow for cross-site comparisons. Reduced EEG files were then exported in European Data Format without resampling. EEG was then analysed via a custom-built MATLAB pipeline. Briefly, EEG files were re-referenced to a Laplacian montage and filtered with second-order Butterworth bandpass filter from 1 to 70 Hz and a second-order 60 Hz notch filter. The resting EEG was then split into segments of 0.5 s duration, and an automated rejection procedure was applied to eliminate segments with excessive ocular, muscular or motion artefact. Segments whose amplitude exceeded 500 μV were automatically rejected. The 0.5 s duration segments with root-mean-squared amplitude (calculated with MATLAB’s rms command) or line length (calculated as $\sum \left|\frac{d}{dt}x(t)\right|$) values greater than the mean + 2 × standard deviation over the entire record were automatically rejected. Non-overlapping segments of non-rejected EEG of 4 s duration were then identified and used for subsequent calculations. This rejection procedure retained a median of 4 min (34%) of the resting EEG record (range: 0.53–6.93 min; 8.57–69.22%). In the time domain, the mean EEG amplitude, standard deviation of the EEG amplitude, kurtosis of the EEG amplitude and skewness of the EEG amplitude were computed for each channel for each 4 s segment. For each channel in each 4 s segment, a power spectral density estimate was obtained and used to calculate 1–4 Hz power (delta), 4–8 Hz power (theta), 8–13 Hz power (alpha), 13–25 Hz power (beta) and 1/f slope. Similarly, for each channel in each 4 s segment, the following power ratios were calculated: theta/delta, alpha/delta, beta/delta, alpha/theta, beta/theta and beta/alpha. To allow for increased reproducibility of our qEEG analysis, we have made our EEG analysis pipeline available to the public on GitHub (github.com/mulcaheyp). Representative values for each qEEG parameter were determined by taking the median of values for single parameters obtained over all channels and all 4 s segments. To facilitate comparisons of band power values, all band power values were log transformed prior to analysis.

### Statistical analyses

Non-parametric Mann–Whitney U-tests were used to assess group differences due to non-normality and unequal variances between the CDD and TD groups. For each of the CDD and TD groups, linear regression was used to characterize associations between the EP/qEEG parameters and participant age, with participant age log-transformed (log_10_) to account for the positively skewed distribution. For the CDD group, linear regression was used to assess associations between the EP/qEEG parameters and each of the CDD severity measures (CSS/MBA). Additional multivariable analyses were conducted to determine the added predictive value of combining qEEG and EP parameters into a single model of severity. For this analysis, Pearson correlations were used to determine the qEEG, VEP and AEP parameters with the strongest linear relationship with each severity measure. The highest-correlated qEEG measure was then entered into a regression model with the highest-correlated VEP or AEP parameter. Improvement in model fit was assessed using ANOVA. Results were considered statistically significant at *P* < 0.05.

The agreement between baseline and Year 1 EPs was characterized using intraclass correlation coefficients (ICCs). ICC estimates were computed using a two-way mixed effects model with the absolute agreement and single measures. ICCs were interpreted as poor (<0.5), moderate (0.5–0.75), good (0.75–0.9) and excellent (>0.9).^30^ Statistical analyses were conducted in IBM SPSS Statistics 26 (IBM, Armonk, NY, USA) with the exception of the multivariable analyses, which were conducted in R (V. 3.5.0, R Core Team, Vienna, Austria).

### Data availability

Data are available upon request from the corresponding author.

## Results

### Evoked potentials

#### Group comparisons

**Figure 2:**
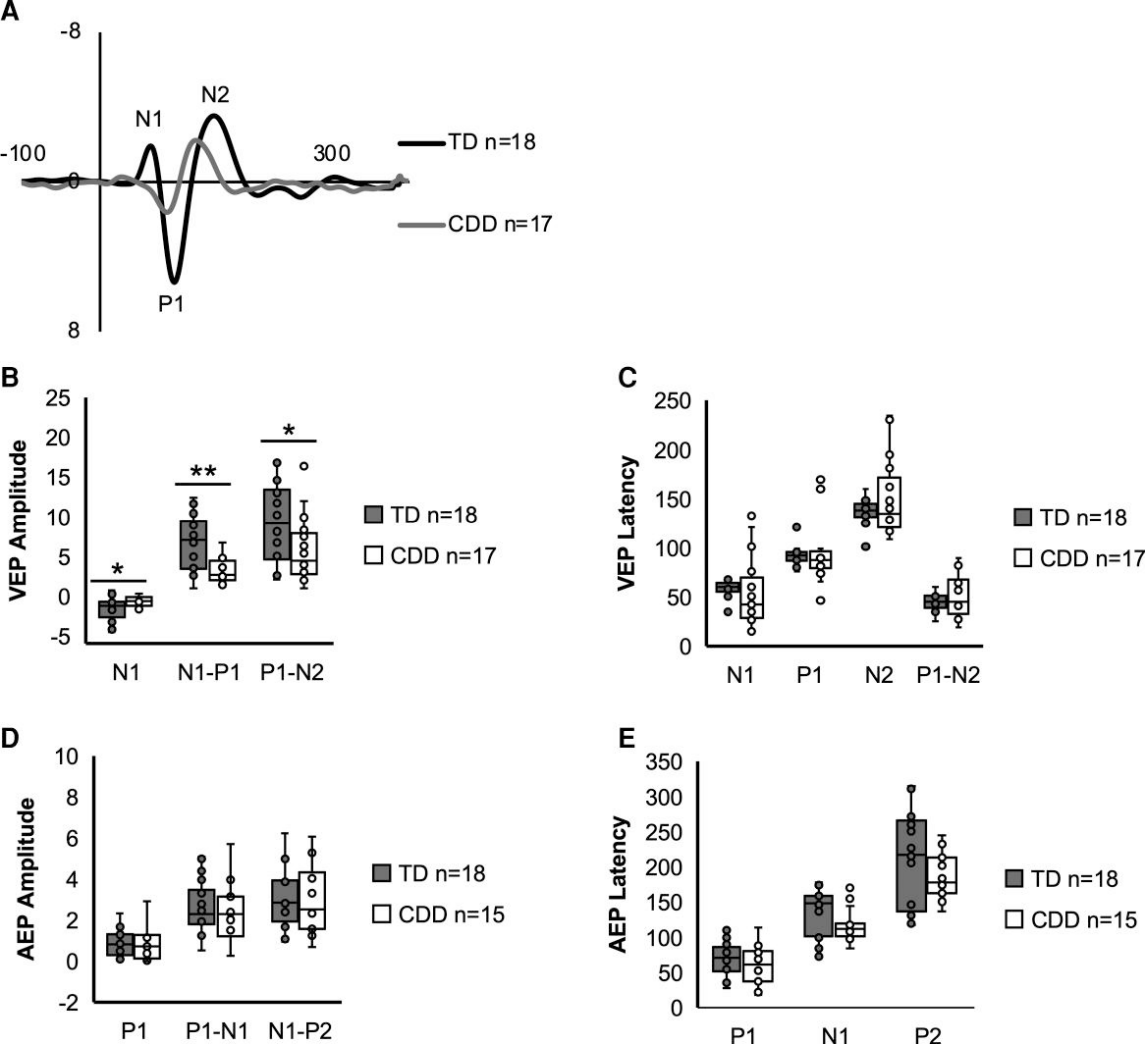
**Group comparison of VEP and AEP.** Grand average VEP waveforms for the TD (shown in grey) and CDD (shown in white) groups at electrode Oz (**A**). Box plots showing the median value and inter-quartile range for the amplitude and latency of the VEP components in TD (shown in grey) and CDD (shown in white) participants (**B** and **C**). VEP N1 (*U* = 216.0, *P* = 0.038), N1–P1 (*U* = 63.0, *P* = 0.002) and P1–N2 (*U* = 90.0, *P* = 0.038) amplitudes were reduced in participants with CDD compared with TD participants. Box plots showing the median value and inter-quartile range for the AEP components in TD (shown in grey) and CDD (shown in white) participants (**D and E**). There were no differences in the amplitude or latency of the AEP components between the two groups. The grand average AEP is not pictured due to age-related changes in peak latency, which obscured comparisons of peak amplitudes. For AEPs from individual participants, see Fig. 6. Statistical analyses were performed using the Mann–Whitney U-tests. **P < 0.01, *P < 0.05.

Participants with CDD had smaller VEP amplitudes compared with TD participants: N1 (*U* = 216.0, *P* = 0.038), N1–P1 (*U* = 63.0, *P* = 0.002) and P1–N2 (*U* = 90.0, *P* = 0.038) (Fig. 2A and B). There were no group differences in the latency of the VEP components (*P* > 0.05) or in the amplitude or latency of the AEP components (*P* > 0.05; see Fig. 2C–E).

#### Associations with age

The latency of the AEP declined with age in TD participants (P1: *β* = −0.773, *P* < 0.001; N1: *β* = −0.868, *P* < 0.001; P2: *β* = −0.702, *P* = 0.001), as expected based on the typical maturation of the AEP.^31^ The latency of the AEP was not significantly associated with age in participants with CDD (*P* < 0.05). No aspects of the VEP were associated with age for CDD or TD participants (*P* < 0.05). Clinical severity was not associated with age for the participants in the analysis of the VEP and/or AEP (CSS: *β* = −0.102, *P* = 0.669; MBA: *β* = −0.066, *P* = 0.781).

#### Associations with severity: baseline

**Figure 3:**
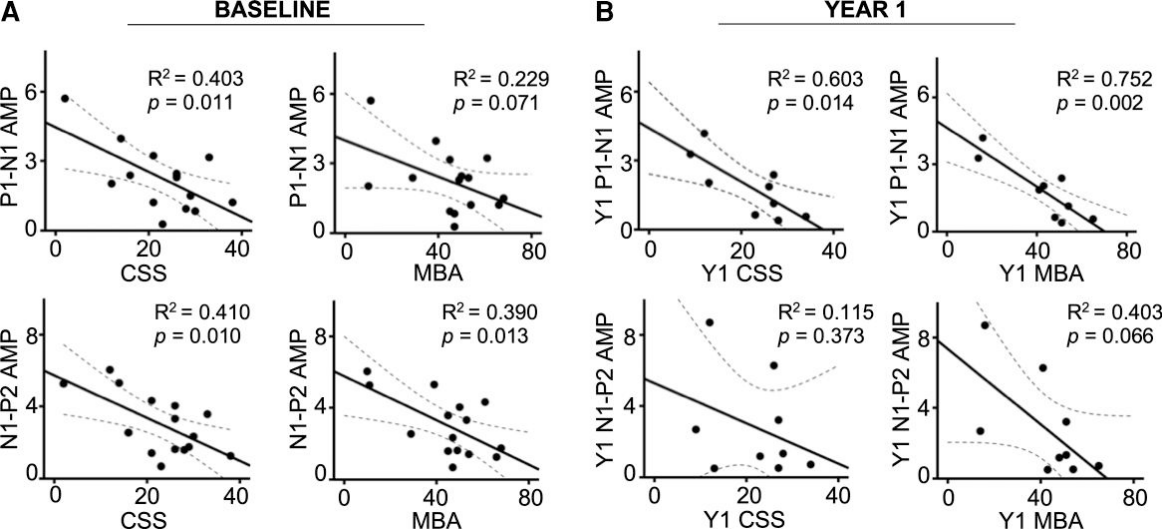
**Associations between AEP amplitude and clinical severity.** Associations between clinical severity and AEP P1–N1 and N1–P2 amplitudes at baseline (*n* = 15; **A**) and Year 1 (*n* = 8; **B**). Statistical analyses were performed using linear regression. Dotted lines represent 95% confidence intervals.

For CDD participants, AEP P1–N1 and N1–P2 amplitudes were negatively associated with clinical severity (Fig. 3A). The association between AEP P1–N1 amplitude and severity was specific to the CSS (*β* = −0.635, *P* = 0.011). AEP N1–P2 amplitude was associated with both the CSS (β = −0.640, *P* = 0.010) and MBA (β = −0.624, *P* = 0.013). AEP N1 latency was also associated with CSS, with decreasing latency with greater severity (β = −0.521, *P* = 0.044). In contrast, no aspects of the VEP were significantly associated with severity measures (*P* > 0.05).

#### Associations with severity: Year 1

Consistent with the results of the baseline analyses, Year 1 AEP amplitude was associated with Year 1 clinical severity, with declining amplitude with greater severity. At Year 1, this pattern was specific to P1–N1 amplitude, which was associated with severity as measured by the CSS (*β* = −0.776, *P* = 0.014) and MBA (*β* = −0.867, *P* = 0.002). N1–P2 amplitude was not significantly associated with either clinical measure (see Fig. 3B). Consistent with the results of the baseline analyses, there were no associations between the VEP and clinical severity in Year 1.

#### Associations with seizure frequency and medications: baseline

For participants included in the analysis of the VEP and/or AEP (*n* = 24), seizure frequency was associated with overall severity on the CSS (*β* = 0.671, *P* < 0.001) and MBA (*β* = 0.473, *P* = 0.020). There were no associations between seizure frequency and the VEP parameters (*P* > 0.05). Several aspects of the AEP were associated with seizure frequency including N1 latency (*β* = −0.594, *P* = 0.020), P1 (*β* = −0.600, *P* = 0.018) amplitude, P1–N1 amplitude (β = −0.622, *P* = 0.013) and N1–P2 amplitude (β = −0.572, *P* = 0.026). Given some of these features were also associated with overall severity (CSS/MBA), a hierarchical regression analysis was conducted to examine the independent contribution of seizure frequency after accounting for the effect of severity. CSS was entered at Step 1 and seizure frequency at Step 2 of the hierarchical analysis. The results indicated that seizure frequency did not account for a significant proportion of the variance in AEP N1 latency, P1–N1 amplitude or N1–P2 amplitude over and above the variance accounted for by CSS (*P* > 0.05). There were no associations between the VEP or AEP parameters and the number of medications or the presence or absence of benzodiazepines (*P* > 0.05).

### Quantitative EEG

#### Group comparisons

**Figure 4:**
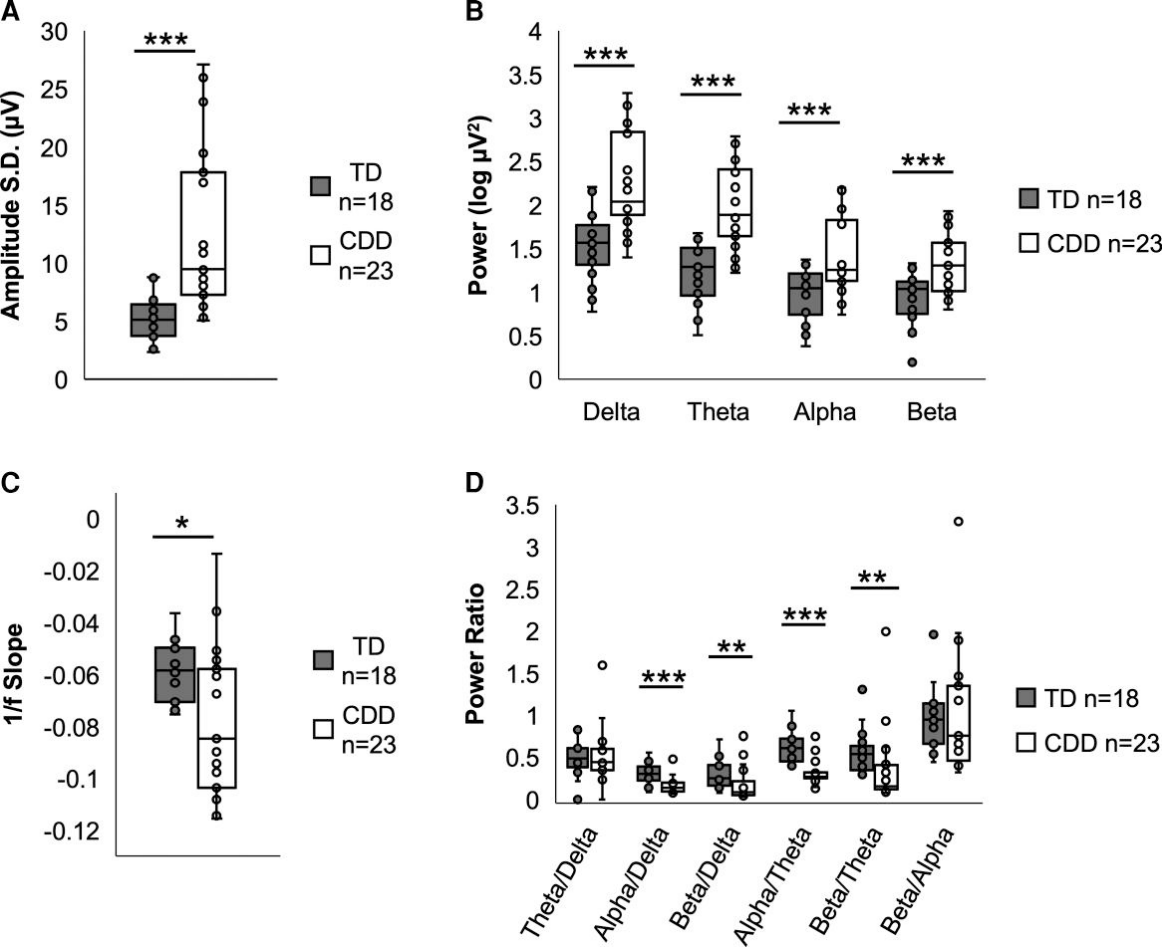
**Group comparison of qEEG parameters.** Box plots showing the median value and inter-quartile range for qEEG parameters for TD (shown in grey) and CDD (shown in white) participants. CDD participants had higher amplitude standard deviation (*U* = 376.0, *P* < 0.001) (**A**), and higher delta, theta, alpha and beta power (*U* = 364.0, 372.0, 328.0, 328.0, respectively, all *P* ≤ 0.001) compared with the TD group (**B**). CDD participants also had more negative 1/f slopes (*U* = 111.0, *P* = 0.012) (**C**), and lower alpha/delta, beta/delta, alpha/theta and beta/theta power ratios compared with TD participants (*U* = 64.0, 96.0, 33.0, 93.0, respectively, all *P* < 0.005) (**D**). Statistical analyses were performed using the Mann–Whitney U-tests. (***P < 0.001, **P < 0.01, *P < 0.05).

The EEG of participants with CDD had a higher global amplitude standard deviation relative to TD participants (*U* = 376.0, *P* < 0.001). Consistent with this observation, participants with CDD had higher delta, theta, alpha and beta power compared with the TD group (*U* = 364.0, 372.0, 328.0, 328.0, respectively, all *P* ≤ 0.001). Participants with CDD had more negative 1/f slopes than TD participants (*U* = 111.0, *P* = 0.012). Finally, participants with CDD had lower alpha/delta, beta/delta, alpha/theta and beta/theta ratios than TD participants (*U* = 64.0, 96.0, 33.0, 93.0, respectively, all *P* < 0.005; see Fig. 4).

#### Associations with age

**Figure 5:**
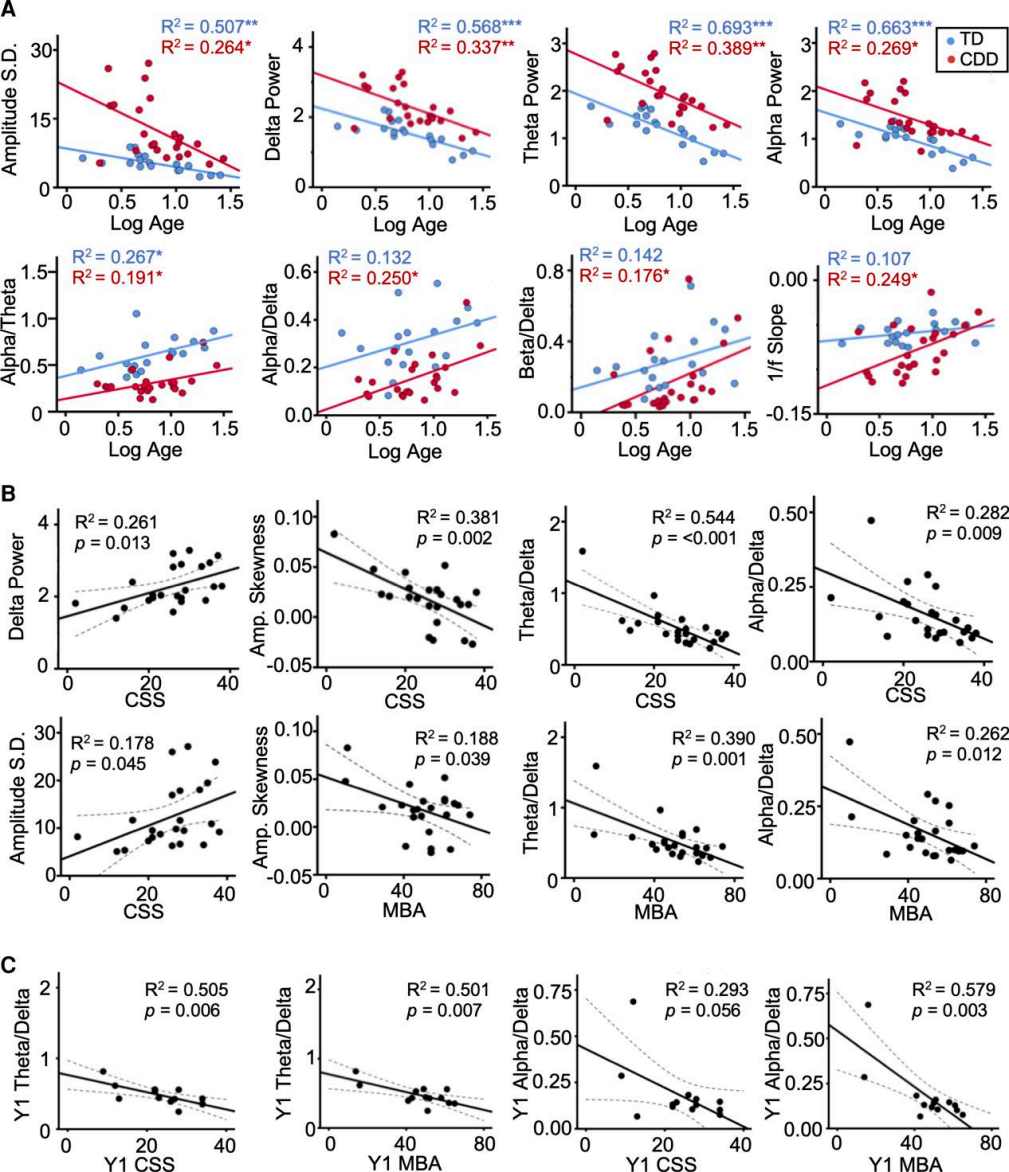
**Associations of qEEG with age and clinical severity.** Associations between qEEG features and age (**A**), qEEG features and clinical severity at baseline (**B**) and qEEG features and clinical severity at Year 1 (**C**). Power is expressed as log μV^2^. Statistical analyses were performed using linear regression. Dotted lines represent 95% confidence intervals. (***P < 0.001, **P < 0.01, *P < 0.05.)

Consistent with established patterns of EEG during development,^32^ amplitude standard deviation, delta power, theta power, alpha power and beta power all decreased with age in the TD cohort (*β* = −0.712, −0.754, −0.833, −0.814, −0.736, respectively, all *P* < 0.001). In the TD cohort, the alpha/theta ratio increased with age (*β* = 0.517, *P* = 0.028). The CDD cohort recapitulated the associations between amplitude standard deviation, delta power, theta power, alpha power and alpha/theta ratio and age (*β* = −0.514, −0.518, −0.598, −0.518, 0.437, respectively, all *P* < 0.05; Fig. 5A). Notably, the CDD cohort did not recapitulate the association of beta power and age (*β* = −0.315, *P* = 0.143). Moreover, associations for alpha/delta ratio, beta/delta ratio and 1/f slope and age were specific to the CDD cohort (*β* = 0.500, 0.420, 0.499, respectively, all *P* < 0.05; Fig. 5A). As with the EP participants, age was not associated with clinical severity for CDD participants in the qEEG analysis (CSS: *β* = −0.030, *P* = 0.892; MBA: *β* = −0.004, *P* = 0.986).

#### Associations with severity: baseline

Within the CDD group, amplitude skewness, theta/delta ratio, alpha/delta ratio, amplitude standard deviation and delta power were associated with clinical severity (Fig. 5B). Amplitude skewness was associated with the CSS (*β* = −0.617, *P* = 0.002) and MBA (*β* = −0.434, *P* = 0.039). The theta/delta ratio was associated with the CSS (*β* = −0.738, *P* < 0.001) and MBA (*β* = −0.625, *P* < 0.001). Similarly, the alpha/delta ratio was associated with the CSS (*β* = −0.531, *P* = 0.009) and MBA (*β* = −0.512, *P* = 0.012). The associations between severity and amplitude standard deviation (*β* = 0.422, *P* = 0.045) and delta power (*β* = 0.511, *P* = 0.013) were specific to CSS.

#### Associations with severity: Year 1

Consistent with the analyses of the baseline data, the theta/delta ratio was associated with clinical severity at Year 1 as measured by CSS (*β* = −0.711, *P* = 0.006) and MBA (*β* = −0.708, *P* = 0.007). Alpha/delta ratio, beta/delta ratio and alpha/theta ratio were additionally associated with severity at Year 1, but these associations were specific to the MBA (Y1 alpha/delta and MBA: *β* = −0.761, *P* = 0.003; Y1 beta/delta and MBA: *β* = −0.634, *P* = 0.020; Y1 alpha/theta and MBA: *β* = −0.569, *P* = 0.042; see Fig. 5C).

#### Associations with seizure frequency and medications: baseline

For participants included in the analysis of the qEEG, seizure frequency was associated with overall severity on the CSS (*β* = 0.717, *P* < 0.001) and MBA (*β* = 0.503, *P* = 0.014). Seizure frequency was associated with several qEEG parameters, specifically alpha/delta ratio (*β* = −0.705 *P* < 0.001), theta/delta ratio (*β* = −0.445, *P* = 0.033) and amplitude skewness (*β* = −0.642, *P* = 0.001). Given these features were also associated with overall severity (CSS/MBA), a hierarchical regression analysis was conducted to examine the independent contribution of seizure frequency after accounting for the effect of severity. The results of this analysis indicated that seizure frequency did not account for a significant proportion of the variance in alpha/delta, theta/delta, or amplitude skewness over and above the variance accounted for by CSS (*P* > 0.05). There were no associations between the qEEG measures and number of medications (*P* > 0.05) or group differences in the qEEG measures for participants on/off benzodiazepines (*P* > 0.05).

### Composite EP/qEEG model of severity

Further regression analysis was performed to determine if a composite qEEG/EP model would provide a stronger prediction of severity than either measure alone. For CSS, the qEEG parameter with the strongest linear relationship was the theta/delta ratio (*r* = –0.740, *P* < 0.001, *n* = 23). Among the AEP parameters, N1–P2 amplitude had the strongest linear relationship with CSS (*r* = –0.640, *P* = 0.02, *n* = 13). Among the VEP parameters, P1 latency had the strongest linear relationship with CSS (*r* = 0.30, *n* = 14), but the relationship was not statistically significant (*P* = 0.30). The addition of AEP N1–P2 amplitude, VEP P1 latency or any other EP parameter to the theta/delta ratio model did not significantly improve model performance (*P* > 0.05).

For MBA, the qEEG parameter with the strongest linear relationship with severity was also theta/delta ratio (*r* = –0.62, *P* = 0.001, *n* = 23). Among AEP parameters, N1–P2 amplitude had the strongest linear relationship with MBA (*r* = –0.64, *P* = 0.02, *n* = 13). The addition of AEP N1–P2 amplitude or any other AEP parameter to the theta/delta ratio model did not significantly improve model performance (*P* > 0.05).

Among the VEP parameters, N2 latency had the strongest linear relationship with MBA (*r* = 0.38, *n* = 14) though the relationship was not statistically significant (*P* = 0.18). However, the addition of VEP N2 latency to the theta/delta ratio model significantly improved *R*^2^ from 0.49 to 0.65 (*P* = 0.04, *n* = 14; see Supplementary Fig. 2). The addition of any other VEP parameter to the theta/delta ratio model did not significantly improve model performance (*P* > 0.05).

### Baseline: Year 1 comparison

#### Stability and change of EPs from baseline to Year 1

**Figure 6:**
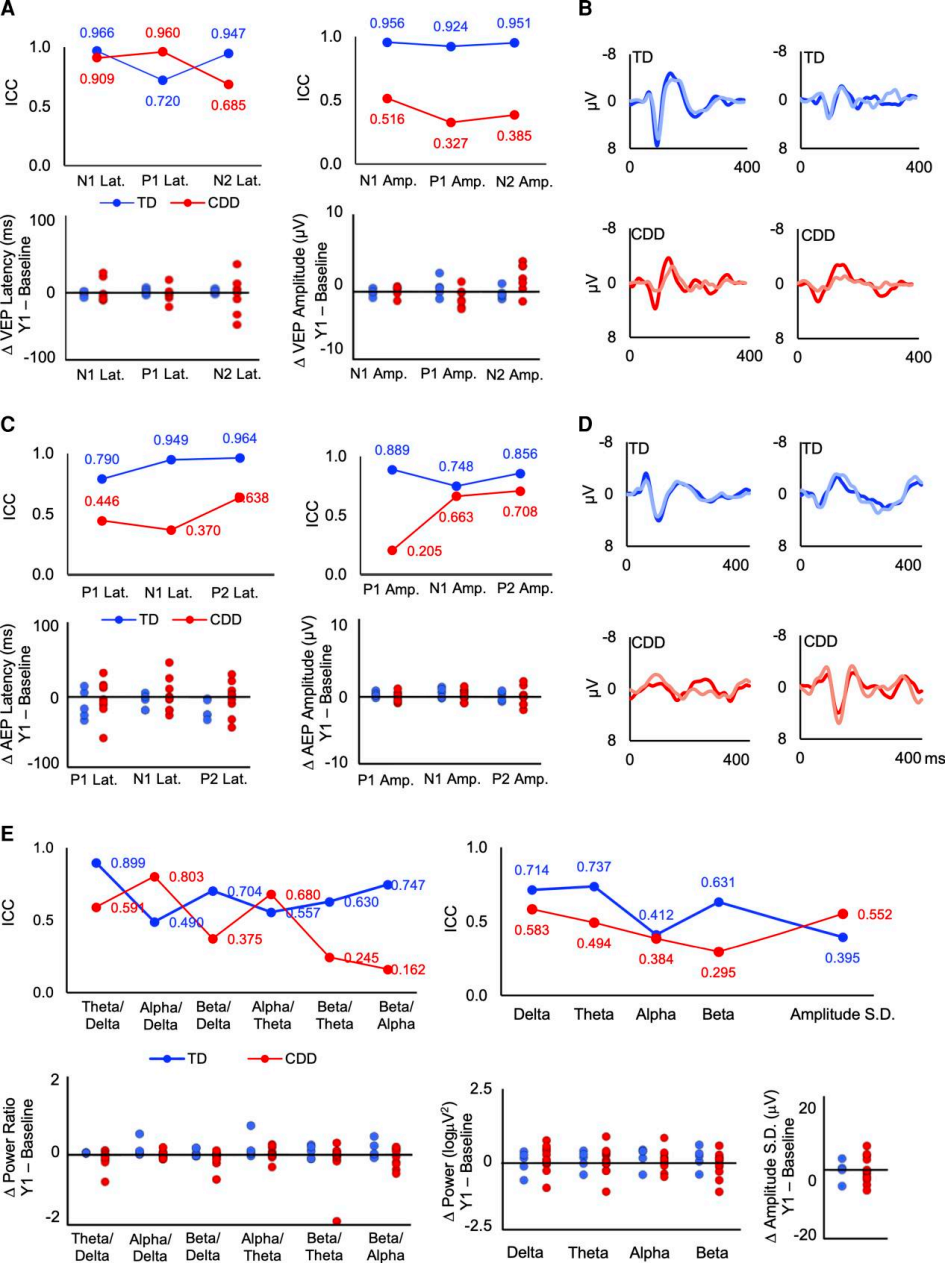
**Change and stability of EP and qEEG features from baseline to Year 1.** ICCs and change plots illustrating change in VEP (**A**), AEP (**C**) and qEEG (**E**) parameters from baseline to Year 1. Change plots represent the difference in Baseline–Year 1 for each TD (blue-left or top graphs) and CDD (red-right or bottom graphs) participant contributing follow-up data. Example VEP (**B**) and AEP (**D**) waveforms for individual TD and CDD participants demonstrating consistency in the EPs, particularly for TD participants.

There was good intersession repeatability in the latency and amplitude of the EPs for TD participants (ICCs = 0.720–0.966; Fig. 6). There was more intersession variability in the EPs for CDD participants, with the exception of VEP N1 and P1 latency, which demonstrated excellent agreement between Baseline and Year 1 (ICCs > 0.9). The latency and amplitude of the AEP components demonstrated moderate agreement (ICCs = 0.531–0.718). VEP amplitude demonstrated poor agreement (ICCs < 0.5). Data from a third (Year 2) visit were available in six participants with CDD. Visual exploration of these data suggests that the pattern at Year 2 was consistent with that for Year 1 such that the responses were consistent for some participants and components and not others (see Supplementary Fig. 4).

#### Stability and change of qEEG

At the group level, the most stable qEEG features for TD participants were theta/delta (ICC = 0.899) and amplitude skewness (ICC = 0.907). Theta power, alpha/delta power, 1/f slope, amplitude standard deviation and mean amplitude demonstrated poor agreement (ICCs = 0.288–0.490). All of the other qEEG features were moderately consistent for the TD group (ICCs = 0.557–0.747). Power ratios were among the more consistent qEEG features for CDD participants, especially alpha/delta ratio (ICC = 0.803). Alpha/theta ratio, theta/delta ratio, delta power and amplitude standard deviation were moderately consistent (ICCs = 0.552–0.680). The other qEEG features demonstrated poor agreement for the CDD group (ICCs < 0.5). As illustrated in Fig. 6C, several of the qEEG parameters demonstrated stability for many of the TD and CDD participants at the individual level. Particularly for the power ratios, the ICC estimates were likely influenced by the one or two participants with more notable change.

## Discussion

The current study characterized abnormalities in visual and auditory EPs and qEEG in individuals with CDD and quantified associations between these features and measures of disease severity. Overall, analyses of the EPs revealed gross attenuation of the VEP in individuals with CDD compared with TD participants whereas the AEP did not differ significantly between groups. qEEG analyses revealed significant differences between CDD and TD participants. Across both EPs and qEEG features, a number of features were associated with clinical severity in CDD and many of these features were reproducible in follow-up recordings at 1 year.

The finding of reduced VEP amplitude in CDD mirrors the pattern observed in individuals with Rett syndrome^13,15^ and echoes the disruption of cortical excitation and inhibition associated with these disorders.^33,34^ Importantly, these group differences remain even when attention to the stimulus is controlled (see Supplementary material and Leblac *et al*.^13^), excluding the possibility that the reduced response is simply due to reduced attention within the clinical groups. The absence of a group difference in the AEP in the current data set is perhaps surprising, particularly considering the notable group differences in the VEP. As a number of CDD participants had to be excluded due to the absence of an identifiable AEP N1 peak (*n* = 8), it is difficult to make strong conclusions regarding AEP abnormalities in CDD based on the current analysis. Future work employing an alternative analysis approach such as template matching is needed for providing a more inclusive description of EPs in CDD (see the ‘Limitations’ section).

While the VEP was dramatically different between the CDD and TD groups, there was no association between the VEP and severity. This contrasts with the pattern observed in individuals with Rett syndrome^15^ as well as animal models of CDD.^35^ As cortical visual impairment (CVI) is a significant clinical feature in CDD,^1,2,7,8,36^ it is possible that the cortical disruption that leads to CVI dramatically alters the VEP in individuals with CDD such that there is no gradation with overall severity. VEP latencies were not abnormal in the current study suggesting the optic pathways were intact,^37^ but the dramatic change in amplitude suggests primary and secondary visual cortical changes as would be expected in CVI. It would be intriguing to compare the amplitudes of the VEP to a CVI scale, as one may predict that the level of CVI would be associated with the change in amplitude of the response. The severity scales employed here (CSS/MBA) were developed for Rett syndrome and do not consider disturbances in visual function. A CDD-specific scale would have been ideal, and indeed, research is now being conducted to examine EPs in the context of a CDD-specific scale that considers aspects of visual function (*NINDS U01-NS114312-02*).

In contrast, the AEP waveforms of CDD participants were similar to the TD participants, but lower amplitudes of the P1–N1 (or N1–P2) did statistically correlate with the severity scales (MBA and CSS). This correlation was reproducible, being present also in the Year 1 data, even with fewer participants. Our AEP findings are similar to the findings in Rett syndrome^15^ suggesting that the AEP could be a robust biomarker of generalized cortical dysfunction across a number of developmental encephalopathies.

Group comparisons of qEEG measures revealed significant abnormalities in a number of global EEG features in individuals with CDD. Compared with TD participants, participants with CDD displayed greater amplitude standard deviation, greater power across all frequency bands, and more negative 1/f slopes. These gross abnormalities in the qEEG match with clinical interpretation of the EEG in CDD.^1,38,39^ Participants with CDD also had lower power ratios indicating increased power in lower versus higher frequency bands when compared with TD participants. Within the CDD group, multiple qEEG parameters correlated with clinical severity. The global qEEG parameter with the strongest association to clinical severity was the theta/delta ratio. EEG power spectral anomalies, including altered band powers and 1/f slope, are characteristic findings in neurogenetic disorders such as Rett syndrome^18^ and Angelman syndrome.^14^ Altered EEG power spectral features are also a characteristic feature of animal models of CDD.^40^ Intriguingly, we found that many qEEG parameters were negatively associated with age, including delta, theta and alpha powers. While this observation recapitulates a typical developmental trajectory for EEG power, it is in apparent conflict with Roche *et al*.’s^18^ observation of a positive association between delta power and age in individuals with Rett syndrome. This apparent discrepancy may occur either because there are genuine differences between the neurodevelopmental physiology of Rett syndrome and CDD or because our study uses global qEEG features, while Roche *et al*.^18^ used qEEG features derived from channels in a frontal lobe region of interest. Overall, our finding of global qEEG ratios correlating with severity scores reinforces the notion that EEG may be a good biomarker for disease severity.

Since both qEEG and EP measures were associated with severity, we attempted to determine if a multivariable model would better predict severity than either measure alone. The addition of an AEP or VEP parameter to the qEEG model (i.e. the theta/delta ratio model) did not significantly improve the CSS model. Intriguingly, for the MBA model, the addition of VEP N2 latency to the qEEG model did improve model performance. Overall, the AEP parameters correlated more strongly with theta/delta ratio compared with the VEP parameters (see Supplementary Fig. 3). This, combined with the multivariable regression findings, may suggest that the AEP parameters and the theta/delta ratio explain similar or redundant variation in the severity scores of the current cohort. On the other hand, the VEP parameters, specifically N2 latency, may account for different variations in severity scores than theta/delta ratio, giving rise to improved performance of a combined EP/qEEG model. This finding hints at the possibility of developing a composite electrophysiological biomarker that is more sensitive to change than any individual feature. Future research should explore this approach further, especially with CDD-specific severity scales that account for CVI.

Epilepsy is a defining feature of CDD with daily seizures occurring in more than half of affected individuals.^41^ In the current cohort, greater seizure frequency was associated with greater global severity (CSS/MBA). Several EP and qEEG features were also associated with seizure frequency, including AEP amplitude, alpha/delta ratio and amplitude skewness. When controlling for overall severity, the association between these factors and seizure frequency was no longer significant. This suggests that the association between the EP/qEEG features and seizure frequency could have been driven by effects of severity rather than the seizures themselves and supports the notion of EP and qEEG parameters as quantitative measures of encephalopathy. However, given the restricted sample size and power to detect smaller effects, ongoing work would be useful to further understand the association between seizure variables and EPs/qEEG parameters in individuals with CDD.

A major need in developing biomarkers is demonstrating clinical reproducibility of the measures. In this study, ICCs were used to characterize repeatability in the EP and qEEG parameters for the subset of TD and CDD participants with follow-up data at Year 1. Year 1 data were only acquired in a few TD participants given the known reproducibility of EPs in TD individuals with the exception of predictable, developmental changes.^23–26^ Due to small samples sizes available for Year 1 analyses for both the TD and CDD groups, stability in the EP and qEEG parameters was additionally characterized using difference plots with change from baseline to Year 1 plotted for each individual participant. Overall, this analysis revealed excellent intersession repeatability in the EPs for TD participants. The timing and amplitude of the EPs were not as consistent for CDD participants, although many parameters including VEP latency and AEP amplitude demonstrated either moderate or excellent agreement. Changes in the VEP and AEP parameters among the CDD participants cannot be attributed to changes in clinical severity as the clinical scores for all participants remained stable across the two time points (see Supplementary Table 1). More likely, the inconsistency in the EPs between the session stems from differences in arousal and/or the extent of movement and other EEG artefacts between sessions. Indeed, the EP parameters with the least repeatability among CDD participants were VEP amplitude, which is known to be affected by attention.^37,42^ The finding of consistent EPs for the TD participants is encouraging that the variability observed for CDD participants can be reduced, perhaps by ensuring attention to the stimulus as well as other behavioural and technical factors remain constant between sessions. Similar to the EPs, the qEEG parameters were generally more stable for the TD (versus CDD) participants. However, unlike the EP parameters, several of the qEEG parameters (e.g. mean amplitude, 1/f slope) were not consistent in either the TD or CDD groups. The more consistent factors are likely the best biomarkers. Notably, the theta/delta ratio, which was the strongest predictor of clinical severity, was one of the most stable qEEG measures between baseline and Year 1 measurements, underscoring its potential utility as a biomarker of treatment response.

### Limitations

There are a number of limitations in this study that need to be addressed by future work. Approximately one-third of participants with CDD had to be excluded from the analysis of the VEP and/or AEP due to excessive artefact or absence of identifiable components above the noise level. In some participants, the absence of identifiable components may be due to residual artefact or technical factors (e.g. poor signal at channels of interest, inaccurate cap placement, etc.). In other participants, a relatively flat waveform may reflect severe cortical dysfunction. As this is the first study of EPs in CDD, we took a conservative approach of excluding these participants. Ongoing work is urgently needed to identify novel methods for reducing artefact, improving signal-to-noise ratio, and quantifying EPs in this population. Furthermore, only a subset of participants contributed Year 1 data, resulting in a low sample size for the repeatability analyses. Future work with a larger sample is needed for a more comprehensive understanding of the stability and change in EPs and qEEG in individuals with CDD. To inform future clinical trials, this work will require a large enough sample to establish boundaries for what may be considered normal variation versus clinically significant change, and these ranges will also need to account for expected developmental change. Only one of the five study locations employed eye tracking to confirm attention during the VEP paradigm. At the other four sites, the VEP continued even when participants were gazing away from the stimulus, and therefore, attentional effects are likely an additional source of noise in the current data set. Future studies should employ eye tracking for all participants to control for effects of attention. Visual and hearing tests were not performed and, therefore, it is not known how vision and hearing problems may have affected the results. Finally, we focused on global changes in EEG to test the hypothesis that patients with CDD have a diffuse encephalopathy and global EEG parameters would be most sensitive to patient severity. However, global EEG parameters may fail to capture region or network-specific changes associated with CDD severity so subsequent work focusing on regional differences may improve these findings. In addition, this study used basic descriptive EEG features. There are a variety of qEEG techniques available, including coherence and network-based measures and these avenues are potential extensions of the work presented in this study.

## Conclusion

This study compared features of VEPs, AEPs and qEEG in CDD and TD participants, and uncovered important group differences in the VEPs and qEEG. We found that features of the AEPs and qEEG correlate with clinical severity and that these features are reproducible between baseline and Year 1 measurements. The VEP did not correlate with the clinical severity measures used here, possibly due to mediating influences of CVI, which were not captured in these measures. We assessed the validity of combined EP/qEEG models for severity and found that a combined VEP/qEEG model may predict MBA scores better than a single modality model. Together, the findings provide support for the use of EPs and qEEG measures as biomarkers of severity in CDD. Finally, the current study may provide a template for developing such biomarkers for other developmental encephalopathies.

## Supplementary Material

fcac197_Supplementary_Data

## Abbreviations

AEP =: auditory evoked potentials
BCH =: Boston Children’s Hospital
CCH =: Cincinnati Children’s Hospital
CDD =: CDKL5 deficiency disorder
CHOP =: Children’s Hospital of Philadelphia
CSS =: clinical severity scale
CVI =: cortical visual impairment
EP =: evoked potential
ICCs =: intraclass correlation coefficients
MBA =: Motor-Behavioral Assessment
NHS =: Natural History Study
qEEG =: quantitative EEG
TD =: typically developing
UC-CHCO =: University of Colorado/Children’s Hospital Colorado
VEP =: visual evoked potential
VUMC =: Vanderbilt University Medical Center
