# Supplementary material for: Electrophysiological biomarkers of brain function in CDKL5 deficiency disorder
Source: Brain Commun. 2022 Aug 4;4(4):fcac197. doi: 10.1093/braincomms/fcac197 (PMC9374482; doi:10.1093/braincomms/fcac197)
Supplement: fcac197_Supplementary_Data [file fcac197_supplementary_data.docx]

**Supplementary Material**

**Oz**

**Supplementary Figure 1.** Grand average VEP waveforms for TD (*n*=9; median age = 4.7 years) and CDD (*n*=10; median age = 3.6 years) participants from the study location (BCH) employing an attention-contingent VEP paradigm. The results are consistent with those reported in the main manuscript (with VEP averaged across all 5 sites), namely a reduction in VEP amplitude in CDD vs. TD individuals.

**Supplementary Figure 2.** Scatterplots of predicted vs. actual MBA values for the simple regression models with theta/delta ratio (**A**) and VEP N2 latency (**B**) and the multiple regression model with theta/delta ratio and VEP N2 latency (**C**).


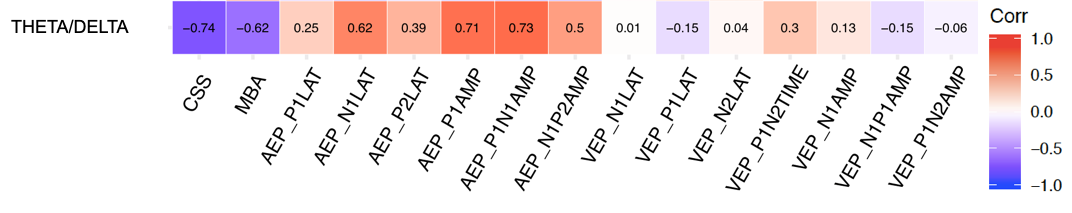


**Supplementary Figure 3.** Correlations between theta/delta ratio and EP parameters. Theta/delta ratio is more strongly correlated with AEP amplitude than with VEP latency. This, combined with the multivariable regression findings, may help to explain why the addition of VEP N2 latency to the theta/delta ration regression model improves MBA model performance, while the addition of AEP amplitude does not.

|  | Age (years) | | | CSS | | | MBA | | |
| --- | --- | --- | --- | --- | --- | --- | --- | --- | --- |
| Participant | Base | Year 1 | Year 2 | Base | Year 1 | Year 2 | Base | Year 1 | Year 2 |
| 1 | 1.9 | 3.4 |  | 26 | 27 |  | 53 | 54 |  |
| 2 | 1.7 | 2.9 |  | 30 | 27 |  | 47 | 51 |  |
| 3 | 2.5 | 3.0 |  | 28 | 34 |  | 53 | 58 |  |
| 4 | 5.1 | 5.7 |  | 16 | 13 |  | 29 | 43 |  |
| 5 | 6.0 | 7.6 | 8.1 | 38 | 34 | 38 | 66 | 65 | 66 |
| 6 | 6.3 | 7.3 |  | 21 | 22 |  | 61 | 49 |  |
| 7 | 6.7 | 8.3 |  | 2 | 9 |  | 11 | 14 |  |
| 8 | 8.2 | 9.0 | 10.3 | 34 | 32 | 35 | 62 | 62 | 74 |
| 9 | 9.7 | 10.8 |  | 33 | 28 |  | 69 | 61 |  |
| 10 | 10.1 | 11.0 | 12.3 | 23 | 23 | 29 | 47 | 50 | 49 |
| 11 | 10.6 | 11.6 |  | 27 | 27 |  | 54 | 52 |  |
| 12 | 10.7 | 11.2 |  | 28 | 22 |  | 45 | 45 |  |
| 13 | 10.9 | 12.2 |  | 36 | 37 |  | 74 | 71 |  |
| 14 | 15.7 | 16.8 | 18.2 | 29 | 28 | 32 | 68 | 51 | 61 |
| 15 | 20.2 | 22.1 | 23.3 | 12 | 12 | 13 | 10 | 16 | 18 |
| 16 | 27.2 | 28.2 | 29.4 | 26 | 26 | 29 | 50 | 41 | 50 |

**Supplementary Table 1.** Age and severity scores for participants who returned for follow-up EPs/qEEG acquisition. Six participants completed a third (Year 2) visit in addition to Baseline and Year 1. **CSS**= Clinical Severity Scale; **MBA** = Motor Behavioral Assessment.

**
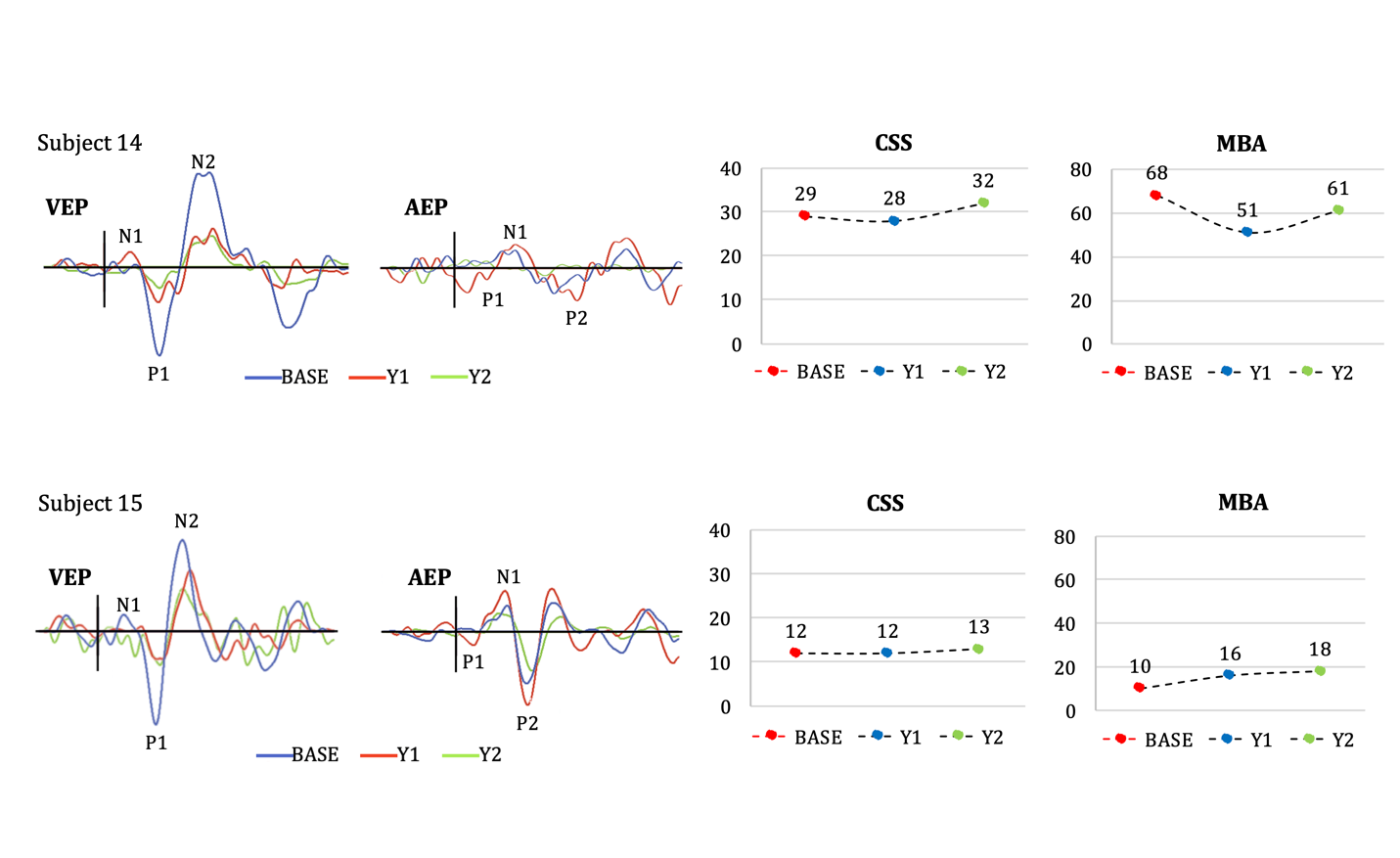
Supplementary Figure 4.** Visual (VEP) and auditory (AEP) evoked potentials and severity scores for two participants contributing data at three timepoints (Baseline, Year 1, and Year 2). Although these participants demonstrated inconsistent VEP amplitudes between Baseline and Year 1, these responses were more consistent from Year 1 to Year 2. The AEP response was relatively reproducible across all three visits for Subject 15 (bottom row). For Subject 14 (top row), the AEP was consistent between Baseline and Year 1, but no N1 peak was observed at Year 2. Clinical scores for these participants remained stable over the duration of the study period.
